# Brain Injury and Mental Health Among the Victims of Intimate Partner Violence: A Case-Series Exploratory Study

**DOI:** 10.3389/fpsyg.2021.710602

**Published:** 2021-10-05

**Authors:** Gunnur Karakurt, Kathleen Whiting, Stephen E. Jones, Mark J. Lowe, Stephen M. Rao

**Affiliations:** ^1^Department of Psychiatry, Case Western Reserve University, Cleveland, OH, United States; ^2^University Hospital Cleveland Medical Center, Cleveland, OH, United States; ^3^Uniformed Services University of the Health Sciences, Bethesda, MD, United States; ^4^Diagnostic Radiology, Cleveland Clinic, Cleveland, OH, United States; ^5^Cleveland Clinic Lou Ruvo Center for Brain Health, Cleveland, OH, United States

**Keywords:** intimate partner violence, traumatic brain injury, brain imaging, mental health, abusive relationship

## Abstract

Intimate partner violence (IPV) survivors frequently report face, head, and neck as their injury site. Many mild traumatic brain injuries (TBIs) are undiagnosed or underreported among IPV survivors while these injuries may be linked to changes in brain function or pathology. TBI sustained due to IPV often occurs over time and ranges in severity. The aim of this case-series study was to explore risk factors, symptoms, and brain changes unique to survivors of intimate partner violence with suspicion of TBI. This case-series exploratory study examines the potential relationships among IPV, mental health issues, and TBI. Participants of this study included six women: 3 women with a history of IPV without any experience of concussive blunt force to the head, and 3 women with a history of IPV with concussive head trauma. Participants completed 7T MRI of the brain, self-report psychological questionnaires regarding their mental health, relationships, and IPV, and the Structured Clinical Interview. MRI scans were analyzed for cerebral hemorrhage, white matter disturbance, and cortical thinning. Results indicated significant differences in resting-state connectivity among survivors of partner violence as well as differences in relationship dynamics and mental health symptoms. White matter hyperintensities are also observed among the survivors. Developing guidelines and recommendations for TBI-risk screening, referrals, and appropriate service provision is crucial for the effective treatment of TBI-associated IPV. Early and accurate characterization of TBI in survivors of IPV may relieve certain neuropsychological consequences.

## Introduction

Intimate partner violence (IPV) is a public health concern that can lead to physical, sexual, and psychological harm (Breiding et al., 2015). It is estimated that one in every four women in their lifetime experience IPV (Sheridan and Nash, 2007; Breiding et al., 2015). Facial, head, and neck injuries are among the most commonly reported injuries by IPV survivors (Sheridan and Nash, 2007; Karakurt et al., 2017). Depending on the severity of violence, survivors frequently reported various neurological symptoms and mental health issues including anxiety, depression, and Post Traumatic Stress Disorder (PTSD; Bonomi et al., 2006; Black, 2011). While some of these neuropsychological disorders maybe related to Traumatic Brain Injury (TBI), i.e., due to changes in brain function or developing brain pathology as a result of external force (Brain Injury Association of America, 2020), these changes might go unnoticed due to the similarity with non-TBI related IPV comorbidities (Kwako et al., 2011). Many mild TBIs are undiagnosed or underreported (Davis, 2014).

The physiological effects of TBI manifest in several different ways, often depending on the mechanism of injury (Banks, 2007; Brain Injury Association of America, 2020). Typical tissue injury involves shearing forces at the junctions between gray and white matter (Kwako et al., 2011). In addition, the axonal stretch may impair the transport of neurotransmitters along the axons of cortical nerve fibers, reducing the operational efficiency and efficacy of neural networks (Henckens et al., 2009). Neuronal death resulting from the TBI may lead to hyper glucose toxicity and could induce post-traumatic epilepsy (Nugent et al., 2014). Trauma also causes neuroinflammation, inducing microglial response, apoptosis, and release of inflammatory cytokines (Yakovlev and Faden, 2004). Reactive oxygen species and nitrogen oxides can have serious effects on inflammatory and neural processes within the central nervous system (Yakovlev and Faden, 2004). TBI often involves coup counter-coup injury where the brain rattles within the skull. These pathological responses are serious and can have long-lasting effects even after mild TBI (Yakovlev and Faden, 2004; Kwako et al., 2011; Davis, 2014).

Default mode connectivity and the default mode network (DMN) are indicative of basal brain activity when the brain is not focusing or attending to any specific task; when the DMN is most active, the brain is not focusing on anything in particular, and indeed DMN suppression is necessary for goal-directed cognition (Anticevic et al., 2012). TBI is associated with increased DMN activity, which is consistent with the decreased attention reported by patients and observed decreased white matter connectivity (Bonnelle et al., 2011). TBI has varied epidemiology, which contributes to its difficult pathophysiology (Gardner and Zafonte, 2016; Giza et al., 2017). TBI is characterized by physical trauma to the brain resulting in significant neurovascular, inflammatory and neurological consequences including cell death and dysfunction, associated with varying degrees of cognitive and behavioral deficits. Mouse models have revealed multifocal axonal injury and microglial reactivity following repetitive mild TBI, as well as cerebrovascular abnormalities and mitochondrial dysfunction associated with reactive oxygen species (Robertson et al., 2007; Shitaka et al., 2011; Bennett et al., 2012; Watson et al., 2013; Hiebert et al., 2015; Fischer et al., 2016; Lynch et al., 2016; Ojo et al., 2016; Khatri et al., 2018). Metabolism seems to play a role in mechanisms of TBI pathology, with synaptic mitochondria sustaining more damage than non-synaptic mitochondria and experiencing more severe oxidative stress and respiratory dysfunction (Kulbe et al., 2017; Hill et al., 2018). Additional research into the implications of TBI on autonomic dysregulation suggests a role in the non-neurological complications of hospital stay and paroxysmal sympathetic hypersensitivity (Takahashi et al., 2015; Hasen et al., 2019; Khalid et al., 2019; Purkayastha et al., 2019). Cerebrovascular response and vascular repair mechanisms are known to affect long-term TBI outcomes due to the effects of hypoperfusion, ischemia, hemorrhage, blood–brain barrier disruption, and edema. For this reason, assessment of cerebral blood flow during acute and chronic TBI treatment is vital for reducing and preempting the effect of secondary injury (Akbik et al., 2016; Salehi et al., 2017). Survivors of IPV suffer from injuries that predominantly affect the neural structures that impede behavioral and decision-making skills including the amygdala, the prefrontal cortex, the hypothalamus, and the hippocampus (Phelps, 2004; Haubensak et al., 2010; Rodrigo et al., 2016; St Ivany and Schminkey, 2016). Neuroanatomical studies reveal that injuries to the amygdala could affect emotional processing, hypothalamic-pituitary-adrenal responses, memory, and flexibility in social learning (Phelps, 2004; Shirazi et al., 2015; St Ivany and Schminkey, 2016; Rodrigo et al., 2016). Injuries to the prefrontal cortex, the hypothalamus, and the hippocampus, are linked with challenges in executive functioning, properly interpreting and planning the sequence of events, and adaptation to the environment (St Ivany and Schminkey, 2016).

The impact of IPV on emotional processing varies across individuals, with distinct patterns of neural circuitry activation demonstrating heterogeneity in symptom internalization among survivors (Sellnow et al., 2020). Structural brain differences among IPV survivors compared to non-victims have been correlated to a history of IPV-related TBI (Daugherty et al., 2020). Mild TBI is known to affect white matter tract integrity as assessed by MRI (Yin et al., 2019). IPV survivors exhibit evidence of altered white matter integrity, as identified by MRI showing decreased fractional anisotropy (FA) in the corpus callosum and corona radiata (Flegar et al., 2011; Valera et al., 2019), but implications on cognitive function are inconclusive and further investigation is needed for more definitive interpretation of results.

However, it is important to note that abnormally reduced FA is more prevalent in individuals diagnosed with mild TBI with PTSD compared to healthy controls and those suffering from mild TBI without PTSD, suggesting a possible direct connection between PTSD and white matter disruption and that PTSD and mild TBI comorbidity may alter the interactions affecting white matter integrity, resulting in potential confounds during interpretation (Davenport et al., 2016; Lepage et al., 2018; Klimova et al., 2019; Santhanam et al., 2019).

Women exposed to physical IPV are more likely to experience psychological distress and cognitive dysfunction relating to brain injury, with one study estimating nearly 75% of survivors may have sustained one or more partner-related brain traumas, where injury severity is negatively associated with cognitive function (i.e., learning, memory, cognitive flexibility) and positively associated with abuse severity and PTSD symptomatology (Woods, 2000; Valera and Berenbaum, 2003; Woods et al., 2008; Davis, 2014; St Ivany and Schminkey, 2016; Iverson et al., 2017; Campbell et al., 2018; Esopenko et al., 2021). Women who experience IPV-related TBI are at a heightened risk of worse long-term psychosocial health outcomes, and while some research shows that TBI severity is related to depression and anxiety but independent of PTSD, many studies suggest improved IPV-specific screening tools are critical for more accurate and effective patient care (Gerber et al., 2014; Iverson and Pogoda, 2015; Goldin et al., 2016; Murray et al., 2016; St Ivany and Schminkey, 2016; Amoroso and Iverson, 2017; Cimino et al., 2019; Iverson et al., 2019, 2020; Smirl et al., 2019; Haag et al., 2019a,b; Liu et al., 2020; Fortier et al., 2021; Meyer et al., 2021).

Women exposed to IPV but independent of PTSD show alterations in brain network connectivity relating to cognitive-emotional control, with the principal involvement of the caudate anterior cingulate, the middle temporal gyrus, the ventral diencephalon, and the left amygdala (Roos et al., 2017). Women with a history of IPV-related PTSD show hyper-activation in the basolateral amygdala and cortical language processing regions in response to the trauma-associated verbal cues, indicative of enhanced processing and hyper-vigilant response to triggering vocabulary compared to healthy controls (Neumeister et al., 2018), with similar findings of the amygdala and cortical activation during the presentation of traumatic visual cues (Neumeister et al., 2017). Functional connectivity analysis also shows increased activation in the insula during cued anticipation of negative events, but the causality of IPV, PTSD, or pre-existing brain function is unclear (Simmons et al., 2008). IPV-related PTSD seems to affect neural flexibility during inhibition, involving difficulty disengaging DMN activation and modulating executive control (Aupperle et al., 2016), as well as displaying hyperactivity and disconnection of limbic sensory systems when processing threat-related emotion and dysregulated the brain activity of the anterior cingulate and prefrontal cortices during pain processing which could drive maladaptive coping mechanisms (Fonzo et al., 2010; Strigo et al., 2010). Women with IPV-related PTSD showed altered activation of the prefrontal cortex, anterior cingulate cortex, and the hippocampus in response to menacing and emotional male–female interaction scenes suggesting impaired social perception of emotionally charged interactions (Moser et al., 2015).

Further research has also shown that increased severity of TBI in IPV survivors is associated with reduced intrinsic functional connectivity between the right anterior insula and posterior cingulate cortex, correlating with greater impairment of the cognitive performance on memory and learning indices (Valera and Kucyi, 2017). Functional MRI scans of IPV survivors with PTSD indicated increased activation of the anterior/middle insula during negative anticipation exercises, which may involve hyperarousal of neural circuitry in these areas (Simmons et al., 2008). On the epigenetic and molecular level, studies have shown a link between IPV exposure and PTSD, and the role of genetic methylation on symptom severity and trauma-induced brain activation patterns (Schechter et al., 2017). Studies often fail to account for the role of TBI in the onset/proliferation of mental health issues among the survivors of IPV (Bonomi et al., 2006; Banks, 2007; Davis, 2014; St Ivany and Schminkey, 2016).

Pinpointing the origin of symptoms is difficult when TBI and IPV happen simultaneously since it could either be due to the psychological effects of violence or physical changes in the brain of the survivor (Kwako et al., 2011). Mental health issues and TBI symptoms sustained over time may be associated with lasting physical, behavioral, and cognitive consequences, yet the survivors may have not sought medical attention (Zieman et al., 2017). Therefore, the aim of this study was to explore risk factors, symptoms, and brain changes unique to survivors of IPV with suspicion of TBI. In this project, we expect to characterize the long-term consequences of head trauma by investigating the signs of TBI in survivors of IPV using advanced neuroimaging biomarkers at the ultra-high-field strength (7T), particularly focusing on signs of chronic or cerebral hemorrhage, disturbances in white matter, and cortical thinning. For this purpose, we compared two groups of women: (1) survivors of IPV who suffered from blunt force to the head and lost consciousness and (2) survivors of IPV who did not report any blunt force to the head. We hypothesize that women with head trauma and episodes of lost consciousness will show mild TBI indicators in the brain scans, sensory disorientation in self-report questionnaires, and mental health symptomology in Structured Clinical Interview (SCID-IV) results.

### Participants

This pilot case-serial study was composed of six female participants. Participants aged from 18 to 65 years were recruited from domestic violence shelters, local community agencies, and organizations in a large city located in The Midwestern United States. Fliers were shared with the agencies and interested participants contacted the research group. All participants are survivors of IPV. The control group included three women who *had never experienced* a blunt force to the head or loss of consciousness. The experimental group included three women who reported having experienced a blunt force to the head and/or loss of consciousness in the past *because of IPV*.

### Methods

Participants signed an informed consent form before data collection. Data were collected in a single 4-hour session. Data collection included filling out self-report questionnaires, completing the SCID-IV (First et al., 1998), and the 7T MRI of the brain. Completing self-report questionnaires took about 45min.

#### Imaging

Participants underwent a brain scan in a Siemens Magnetom 7T MRI scanner (Erlangen, Germany). The TBI imaging protocol on the 7T scanner involved performing a volumetric T1 sequence (MP2RAGE), blood product-sensitive sequence (SWI/GRE), white matter lesion sequence (T2), resting-state fMRI, and diffusion tensor imaging (DTI).

The following scans were performed on all participants:

1. High resolution T1 anatomical scan: 3D MP2RAGE, 192 sagittal slices, resolution: 0.75mm isotropic, TR/TE/TI1/TI2/flip1/flip2=6,000ms/2.99ms/800ms/2700ms/4/5
2. High resolution T2*: 2D GRE, 60–1.5mm thick axial slices, in-plane resolution=0.38×0.38mm, TR/TE/flip=2,290ms/17.8ms/25
3. High resolution SWI: 3D SWI, 144–0.8mm thick axial slices, in-plane resolution 0.49×0.49mm, TR/TE/flip=23ms/15ms/20
4. FLAIR: 45 2mm thick axial slices, in-plane resolution=0.75×0.75mm, TR/TE/TI/flip=9,000ms/124ms/2600ms.
5. Whole brain resting state fMRI: multi-band GE-EPI, 81–1.5mm thick axial slices, in-plane resolution 1.2×1.2mm, TR/TE/flip=2,800ms/21ms/60, multi-band=3 grappa=2, 132 repetitions.
6. Whole Brain HARDI acquisition, SE-EPI, 100–1mm thick axial slices. In-plane resolution=1mm isotropic, TR/TE=10.6s/61.6ms, value of *b*=1000s/mm^2^, 65 diffusion directions, 7 *b*=0 volumes.

#### Self-Report Questionnaires

Participants answered a brief survey to determine how often they may have experienced traumatic brain injury, as well as the severity of the blunt force that resulted in head trauma. Participants also filled out information regarding demographics. Additionally, participants completed psychological measures that are related to mental health and underlying relationship issues. Specifically, participants completed questionnaires assessing PTSD, depression, and anxiety, intimate partner violence, relationship issues, and general life satisfaction. Completing self-report questionnaires took about 45min. Measures were chosen based on their strong conceptual and psychometric qualities included the following:

### Measured Relationship Issues

*Conflict Tactic Scale 2* (Straus et al., 1996) was used to assess intimate partner violence based on partners’ rating of their own and their spouse’s conflict strategies, measuring the intensity and frequency of psychological/verbal and physical conflict. This scale has been shown to have good reliability and validity. *Emotional Abuse Questionnaire* (*EAQ*; Jacobson and Gottman, 1998; Babcock et al., 2000) with 66 items was used to measure verbal and emotional abuse. *Conflicts & Problem Solving Scale* (*CPS*; Kerig, 1996) was used to assess conflict in relationships.

### Measured Mental Health Issues

*Symptom Checklist* (*SCL90*; Derogatis, 1994) was used to assess acute psychopathology. *Patient Health Questionnaire-9* (*PHQ-9*; Kroenke et al., 2001) was used to assess depression. It is a 9-item measure that can be utilized for screening, diagnosing, monitoring as well as assessing the severity. It has good reliability (*a*=0.89). It also has good results from the mental health professional validation interviews. *Perceived Stress Scale* (*PSS*; Cohen, 1986; Cole, 1999) was used to examine the psychological construct of “perceived stress.” It is a 10-item scale. Has satisfactory reliability and validity. *Quick Inventory of Depressive Symptomatology* (*QIDS*; Rush et al., 2003), a self-report scale, was used to assess symptoms of depression. It is a 16-item scale with satisfactory reliability (*a*=0.86). It also has satisfactory construct validity and consistent results with the Hamilton rating scale for depression and inventory of depressive symptomology. *Emotion Regulation Questionnaire* (*ERQ*; Gross and John, 2003) was used to assess how cognitive reappraisal and expressive suppression may affect the mental health of participants. *Buss-Perry Aggression Scale* (*BPAQ*; Buss and Perry, 1992) was used to evaluate how anger and hostility may associate with mental health. *Generalized Anxiety Disorder-7* (*GAD-7*; Spitzer et al., 2006) was used to assess an individual’s tendency to worry excessively. It has 7 items. The psychometric qualities of the scale are satisfactory. The internal reliability is *a*=0.86. The mental health professional validation interviews also produce good results. *Quality of Life Enjoyment and Satisfaction Questionnaire-Short Form* (*Q-LES-Q SF*; Endicott et al., 1993) was used to assess the degree of enjoyment and satisfaction experienced by the patient during the past week. *Modified PTSD Symptom Scale* (*MPSS-SR*; Falsetti et al., 1993) was used to assess posttraumatic stress disorder.

#### SCID

In order to assess Axis-I, mental health disorders as defined by the DSM-IV Structured Clinical Interview for DSM-IV (SCID-IV) was utilized (First et al., 1998). Ph.D. level licensed mental health provider who is trained for SCID-IV administered the interview. Completing the SCID-IV, Axis I took approximately 1.5h. Specifically, the interview included modules related to mood disorders, anxiety disorders, somatoform disorders, and eating disorders. A clinician-administered structured interview was used to reduce the confounds of self-report measures.

### Data Analysis

#### Imaging

The functional connectivity data were pre-processed in the following manner:

1. Cardiac and respiratory signals were measured during scanning and regressed out using RETROICOR as provided by AFNI (Cox and Hyde, 1997; Glover et al., 2000).
2. Retrospective motion correction was performed using SLOMOCO.
3. Spatial filtering with 2D in-plane Hamming filter was used to improve functional contrast-to-noise ratio (Lowe and Sorenson, 1997; Beall and Lowe, 2014).
4. The data were temporally filtered to remove all fluctuations above 0.08Hz (Biswal et al., 1995; Lowe et al., 1998).
5. The DTI data were post-processed in the following manner:

Image series were concatenated, followed by an iterative motion correction (Sakaie and Lowe, 2010) that included updating of diffusion gradient directions (Leemans and Jones, 2009).

#### Functional Connectivity Analysis

##### Seed Region Definition

Because we did not have task-based activation data to define the posterior cingulate cortex (PCC), we used a combination of anatomic information and the resting state data itself to define the seed regions. In each of the right and left hemispheres, we selected a finite region of the PCC, proximal to the retrosplenial cortex, consistent with the Talairach locations reported in reference (Greicius et al., 2003). Using InstaCorr from the AFNI suite, we used the method described in reference (Lowe et al., 2014) to define a 9-voxel seed region in both hemispheres PCC. Z-maps of DMN are then produced in the following manner:

1. A reference time series is calculated from the linearly detrended arithmetic average of the nine pixels in each of the ROIs.
2. The cross-correlation is calculated between the reference time series for ROI and the time series for each voxel in the brain.
3. To account for individual differences in global signal, each correlation map was converted to a Student’s *t* map (Press et al., 1986). For each of the three Student’s *t* maps, the whole-brain distribution was normalized to unit variance and zero mean (Lowe and Sorenson, 1997). The mean and variance from each distribution are used to convert, voxelwise, the Student’s *t* to a z-score.

#### Self-Report

Exploratory univariate analyses were conducted for the self-report questionnaires. Please see Table 1 for means and standard deviations of trending symptoms for women who lost consciousness and not lost consciousness survivors of IPV.

**Table 1:** Relationship characteristics for the participants.

Relationship characteristics	Case I	Case II	Case III	Control I	Control II	Control III
Isolation	83	80	39	81	53	39
Degradation	66	84	49	96	53	47
Sexual abuse	7	17	10	28	15	8
Property damage	26	15	9	20	18	8
Cooperation	23	10	14	6	17	30
Capitulation	44	33	23	12	25	41
Stonewalling	34	18	17	13	19	29
Verbal aggression	27	25	22	N/A	29	25
Physical aggression	10	6	6	6	6	10
Involving child	15	13	14	14	13	13
Stress	14	25	23	22	6	25
Reappraisal	42	18	27	29	25	30
Suppression	7	16	9	22	9	12
Mental health characteristics						
SCID						
Current major depression	0	1	0	0	0	0
Past major depression	0	0	0	0	1	1
Current manic episode	0	0	0	0	1	0
Chronic depression	0	1	0	0	0	0
Anxiety disorder	0	1	0	0	0	0
Panic attacks	0	1	0	0	1	0
Social phobia	0	1	0	1	1	0
Obsessive compulsive	0	0	0	0	1	0
PTSD	0	1	0	1	1	1
Generalized anxiety	0	0	0	0	1	0
Self-report						
Somatization	1.92	1.83	2.67	1.50	2.33	0.50
Obsessive compulsive	1.20	3.60	3.60	1.30	2.30	0.30
Interpersonal sensitivity	0.62	2.69	2.15	1.31	3.69	1.00
Anxiety	0	2.80	1.70	2.30	2.50	0.80
Hostility	0.50	2.17	1.17	0.33	2.67	1.50
Phobic anxiety	0	3.00	2.86	0.57	1.57	0
Paranoid ideation	0.83	1.83	1.50	1.17	2.00	0.67
Psychoticism	0	1.60	1.50	2.20	2.00	0.67
Depression	0	2.22	2.00	1.00	0.33	0.22
Sleep	1.00	4.00	4.00	3.00	1.00	4.00
Appetite	1	2	2	1	1	2
Generalized anxiety	1	12	4	13	3	7

#### SCID

SCID data are presented in Table 1.

## Results

### Case Investigations

#### Loss of Consciousness Case 1

A 59-year-old retired African-American woman with a high school degree reported a history of physical abuse. Violent altercations involved fists, wall, and a refrigerator. Her symptoms included (i) memory problems, such as difficulty spelling and reading, remembering facts and locations, easily getting lost, and having difficulty with speech, (ii) sensory problems such as sensations of numbness, ringing in the ears, vision difficulties, and loss of taste. Imaging review from the radiologist found moderate burden focal WMH without microbleeds.

#### Loss of Consciousness Case 2

29-year-old Caucasian woman with an associates’ degree reported a history of physical and emotional abuse. Violent altercations involved door, wall, and fist. Her symptoms included (i) memory problems such as losing childhood memories and memories of friends, difficulty finding words, (ii) communication problems, including issues with speech, expressing herself and understanding directions, (iii) affect regulation problems, such as being easily aggravated and overwhelmed, and difficulty understanding her own feelings, (iv) sensory problems such as issues with vision, taste, and smell, (v) feelings of disconnection including losing interest in hobbies and relationships, and preferring to be alone. Imaging review from the radiologist found considerable TBI encephalomalacia, gliosis, hemosiderin in bilateral orbitofrontal (R>L), and more superiorly left frontal lobe.

#### Loss of Consciousness Case 3

50-year-old African-American woman with a master’s degree reported a history of physical violence. These violent altercations involved fists, knives, and a beer bottle. Her symptoms included (i) memory problems, such as trouble remembering faces, names, and importance of daily life, (ii) problems with daily functioning, (iii) communication problems such as difficulty expressing opinions, and (iv) sensory problems such as trouble seeing far distances and tasting food. Imaging review from the radiologist found several foci WMH, one questionable susceptibility-weighted imaging (SWI) focus (right frontal lobe).

#### No Loss of Consciousness Control Case 1

A 54-year-old Caucasian woman with a master’s degree reported experiencing long-term physical abuse, combined with emotional abuse including paranoid jealousy, sexual coercion, and manipulation. The relationship ended about 10years ago. Violent altercations involved fits, walls, and a floor. Her symptoms included (i) memory problems, such as searching for words, difficulty remembering names, difficulty collecting thoughts and expressing ideas, (ii) affect regulation problems such as being tight, difficulty expressing emotions and communicating, fear of emotions from herself and others, and (iii) lost interest and energy. Imaging review from radiologists found no microbleeds and mild-moderate White Matter Hyperintensities (WMH).

#### No Loss of Consciousness Control Case 2

34-year-old Asian woman with a bachelor’s degree. She has full-time employment and reported a history of emotional partner abuse. No history of physical altercations was reported. An imaging review from the radiologist found everything normal.

#### No Loss of Consciousness Control Case 3

26-year-old African-American woman with an associate’s degree reported a history of partner abuse. She reported violent altercations involved hitting a wall. Her symptoms included (i) memory problems such as difficulty in remembering things, (ii) problems with executive functioning such as completing tasks, (iii) problems with affect regulation such as being easily hurt and upset, and (iv) having headaches and neck pain. Imaging review from radiologist found single foci WMH and everything else normal.

### Statistical Results

**Figure 1:**
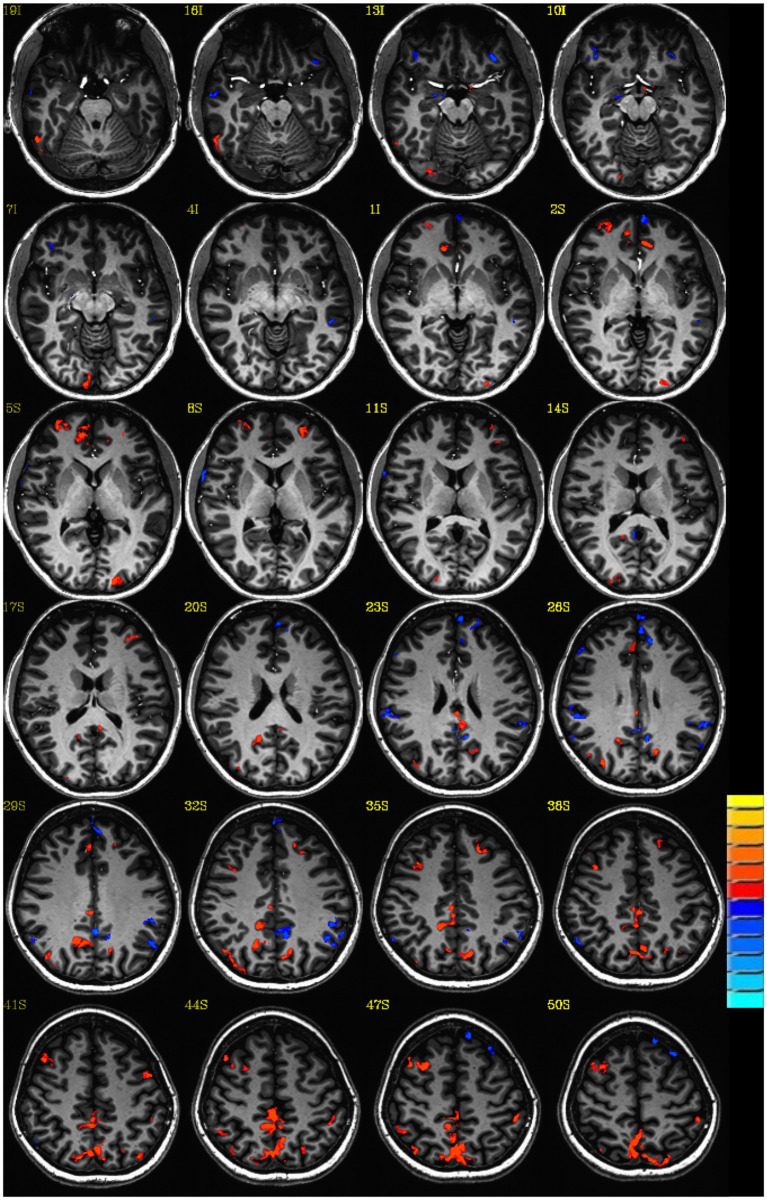
Default mode connectivity: Z-score group difference map: Lost consciousness vs. not lost consciousness head trauma. Single voxel threshold *p*<0.05, cluster requirement >30.

To the extent possible, controls were age-matched to experimental participants to reduce age-related confounds for the imaging analysis. This was necessary since prior studies reveal significant effects of aging including atrophy decreased cortical volume and thickness in cognitively normal older adults (Nugent et al., 2014). All images were transformed into Talairach space for the imaging analysis. An average z-score map was created for each group, head trauma, and no head trauma. The result is a z-score map of connectivity to the PCC commonly referred to in the literature as the DMN (Greicius et al., 2003) indicated a significant difference between the group averaged z-score map for women with no head trauma subtracted from the average z-score map for women with head trauma for DMN. Please see Figure 1.

Results for the self-report questionnaires on personal and relationship characteristics indicated some trends in the dynamics of the abusive relationship, specifically, the tendency of threats and severe violence. Trends of symptoms included hot or cold spells, awakening in the early morning, trouble remembering things, feelings of slowed down, feeling jumpier, or more easily startled, challenges in coping and daily functioning were also observed for women with lost consciousness in contrast with women without lost consciousness (Table 2).

**Table 2:** Mean and Standard Deviation of trending symptoms for women who lost consciousness and not lost consciousness survivors of intimate partner violence.

	Loss of consciousness		No loss of consciousness	
	M	SD	M	SD
Hot or cold spells	4.00	0	1.33	1.53
Awakening in the early morning	4.00	0	0.67	0.57
Trouble remembering things	4.00	0	1.33	1.15
Feelings slowed down	2.33	0.57	1.00	0
Have been jumpier, or more easily startled, since the event(s)	5.00	0	0.67	0.57

## Discussion

Repetitive brain trauma has detrimental effects on IPV survivors’ brain health. Even mild TBI is linked to impairment and reduces the likelihood of full recovery (Cassidy et al., 2004). Our results indicated differences in the neural network functioning among the IPV survivors between head trauma and no-head-trauma groups. Neural connectivity can be described as the interaction patterns observed among the neurons, nerve plexuses, and regions within brain networks (Shirazi et al., 2015). Our results indicated significant differences in resting-state connectivity among survivors of IPV with head trauma. The DMN is helpful in understanding how the brain operates mechanisms that are dealing with a cognitive, physiological, neurobiological system in social cognition, responding to external stimuli, personality expression, decision making, arousal, and ability in perspective-taking (Lee and Lee, 2013). The DMN particularly plays an important role in the deactivation of various brain regions and is deactivated during goal-oriented tasks (Lee and Lee, 2013). Few studies indicated that sleep deprivation and drug abuse can compromise DMN suppression by altering synchronization between brain regions such as the amygdala and precuneus (Phelps, 2004; Haubensak et al., 2010). Survivors of IPV with head trauma displayed higher outflow to DMN regions and cortical arousal which may respond to higher levels of mental activity. Although we did not observe any structural changes in the amygdala regions, we observed that DMN activated during resting state indicating an imbalance between networks. This imbalance between brain networks needs further exploration of deactivation, signal input/output, and cognitive-affective processing, and management of stress-inducing triggers. Our findings on symptoms such as poor appetite, hot or cold spells, awakening in the early morning, trouble remembering things, feelings of being slowed down, feeling jumpier, or more easily startled, challenges in coping and daily functioning can also hint at signs of changes in the survivor’s brain.

Our results found that women who suffered from IPV were presenting symptoms related to depression, anxiety disorders, and PTSD. Past research explains that the physiological symptoms, mechanisms including conditioning of affective responses to trauma and anticipatory anxiety, play a crucial role among survivors of IPV in the progression of PTSD (Brinkmann et al., 2017). An extremely stressful situation that may be life-threatening prompts an autonomic response including the fight or flight responses, functional changes in breathing and heart rate, and fear of having one’s life threatened (Siminski et al., 2021). These emergency-survival responses and physical reactions from the traumatic event can be linked with signals related to the assault situation. When this link is triggered, survivors can re-live the fear response causing intense anxiety to the survivor (Brinkmann et al., 2018; Fox and Shackman, 2019; Awasthi et al., 2020; Siminski et al., 2021). Research suggests the experience of both TBI- and IPV-related concerns are common while controlling for preliminary trauma and comorbid psychological disorders such as PTSD (Etkin and Wager, 2007; Iverson et al., 2017). It is possible that both PTSD and TBI occur more frequently among relationships that have severe violence, or that they may share a similar mechanism. To understand the unique effects of TBI, studies comparing the healthy controls and TBI without IPV are also needed.

Increased awareness of TBI among IPV survivors and increased awareness of the associated adverse health outcomes are crucial (Gerber et al., 2014; St Ivany and Schminkey, 2016; Liu et al., 2020). Our results indicated that certain relationship dynamics such as threats and severe violence are more common among survivors of IPV with head trauma. Findings can be used to improve screening protocols to detect at-risk individuals. Current screening of IPV survivors is inadequate in that they do not sufficiently address the risk of cumulative head trauma on brain health (Goldin et al., 2016; Murray et al., 2016; St Ivany and Schminkey, 2016). Training clinicians on recognizing the signs of mild TBI among survivors of IPV can also be beneficial (Murray et al., 2016).

Despite extensive efforts to identify molecular markers for IPV and TBI, both conditions lack substantiated evidence for specific diagnostic and prognostic biomarkers. Mild TBI does not provide gross pathological changes that can be identified on CT imaging, but clinical validation of peripheral markers for TBI in blood serum and cerebral spinal fluid, samples are underway (Zetterberg and Blennow, 2016; Kerr et al., 2018; Najem et al., 2018; Wang et al., 2018; Thelin et al., 2019). While hair cortisol levels have been associated with IPV and hypothalamic-pituitary-adrenal axis dysfunction, and inflammatory biomarkers like IL-6, IL-1β, and MMP9 in saliva samples of IPV survivors, as well as cardiovascular disease biomarkers may be associated with IPV, these are hardly specific to IPV and can only moderately assist in screening for IPV survivors (Halpern et al., 2017; Shealer et al., 2017; Alhalal and Falatah, 2020). Animal models of TBI have progressed with multiple modes of injury (Xiong et al., 2013), but IPV is understandably more difficult to replicate with animal models due to the social nature of the phenomenon and its potential epidemiological origins (Cordero et al., 2012).

White matter hyperintensities are associated with poor memory performance and more severe PTSD symptoms among veterans, and are correlated with greater TBI severity, and yet correlations with cognitive symptoms are inconclusive and depend on the nature of the disorder (Clark et al., 2016; Tate et al., 2017; van den Berg et al., 2018; Graff-Radford et al., 2019; Berginström et al., 2020; Lippa et al., 2021). The white matter hyperintensities identified by our study may be related to IPV and incidence of TBI, but may also be related to confounds associated with aging; we would require a larger sample size to tease apart the nuances of our findings.

The main limitation of this study is the small sample size and that all the results should be interpreted with caution. The sample of this study includes severely abused women. This is a hard-to-reach population who might need to move frequently for safety and confidentiality concerns. Local shelters are protective of women who stay in shelters. There are multiple layers required to reach out to the participants. For this reason, this exploratory study utilizes a small sample, with a view to paving the way for future studies with larger sample size. Also, it should be noted that due to the condensed timeframe of each session, and the varying stamina levels of the participants, we were unable to maintain a consistent order of administration of phases across subjects. It is also possible that due to memory problems related to TBI, some women might not remember losing consciousness in the past. This might lower the confidence in the control cases as true control cases. We hope to control for these challenges in future studies. Furthermore, in future, studies with larger samples with more power are needed to detect moderate effects. In addition, the biomarkers of mild and severe TBI among survivors of IPV can be used to screen survivors and detect at-risk individuals.

**Table 3:** Mean, Standard Deviation and Voxels on the default mode network regions.

Participants	Left frontal gyrus			Left inferior parietal lobule			Left inferior frontal gyrus			Left middle temporal gyrus			Left posterior cingulate cortex		
	M	SD	V	M	SD	V	M	SD	V	M	SD	V	M	SD	V
Patient 1	2.62	0.28	495	2.82	0.42	765	2.51	0.19	37	2.55	0.19	27	3.07	0.64	982
Patient 2	2.83	0.45	906	2.84	0.46	571	2.49	0.20	17	2.66	0.28	76	3.08	0.66	1,158
Patient 3	3.00	0.57	1,309	3.60	1.01	1,604	2.77	0.39	112	2.95	0.55	367	3.49	0.93	1,618
Mean	2.82			3.09			2.59			2.72			3.21		
SD	0.19			0.44			0.16			0.21			0.24		
Control 1	3.04	0.66	1,015	3.30	0.79	1,269	2.79	0.40	136	3.01	0.66	235	3.66	1.16	1,560
Control 2	3.21	0.85	1,686	3.51	0.94	1,298	3.06	0.65	281	3.18	0.90	669	3.86	1.18	1,424
Control 3	2.75	0.39	403	2.72	0.34	189	2.75	0.37	16	2.57	0.20	36	3.12	0.72	601
Mean	3.00			3.18			2.87			2.92			3.55		
SD	0.23			0.41			0.17			0.31			0.38		
	Right frontal gyrus			Right frontal gyrus			Right inferior frontal gyrus			Right middle temporal gyrus			Right posterior cingulate cortex		
Patient 1	2.71	0.37	412	2.88	0.50	531	2.70	0.30	75	2.56	0.20	75	3.41	1.67	732
Patient 2	2.84	0.45	1848	2.87	0.50	472	2.72	0.36	74	2.76	0.36	155	3.36	1.43	1,069
Patient 3	3.03	0.64	1,501	3.62	1.05	706	2.98	0.52	123	2.98	0.59	175	3.86	1.86	1,227
Mean	2.86			3.12			2.80			2.77			3.54		
SD	0.16			0.43			0.16			0.21			0.27		
Control 1	3.13	0.73	1,212	3.14	0.74	337	3.12	0.73	200	2.69	0.33	124	3.99	1.96	1,144
Control 2	3.28	0.87	1,377	3.33	0.98	420	3.05	0.67	212	3.10	0.72	171	4.60	3.04	1,225
Control 3	2.66	0.31	205	2.65	0.25	150	2.36	0.08	3	2.49	0.14	28	3.53	1.70	542
Mean	3.02			3.04			2.85			2.76			4.04		
SD	0.32			0.35			0.42			0.31			0.54		
	Left frontal gyrus			Left inferior parietal lobule			Left inferior frontal gyrus			Left middle temporal gyrus			Left posterior cingulate cortex		
Patient 1	2.94	0.59	1,076	3.60	0.97	1758	2.68	0.34	280	2.78	0.40	325	4.00	2.25	1,472
Patient 2	3.03	0.60	1,475	3.00	0.56	585	2.87	0.41	35	2.76	0.41	85	3.34	0.96	1703
Patient 3	3.00	0.56	791	3.19	0.80	935	2.75	0.53	85	2.99	0.52	362	3.29	1.30	1,271
Mean	2.99			3.27			2.77			2.84			3.55		
SD	0.05			0.31			0.09			0.13			0.40		
Control 1	3.27	0.92	1,480	3.83	1.32	1850	3.14	0.66	239	3.20	0.88	373	4.22	2.37	1736
Control 2	3.58	1.19	3,703	3.58	0.95	1,530	3.47	0.95	1,054	3.09	0.74	945	4.55	3.49	1,691
Control 3	2.78	0.44	1,121	3.00	0.57	907	2.63	0.28	236	2.82	0.49	576	3.28	2.04	443
Mean	3.21			3.47			3.08			3.04			4.01		
SD	0.40			0.42			0.42			0.19			0.66		
	Right frontal gyrus			Right inferior parietal lobule			Right inferior frontal gyrus			Right middle temporal gyrus			Right posterior cingulate cortex		
Patient 1	2.79	0.46	701	3.12	0.70	808	2.75	0.36	124	2.68	0.31	140	3.79	1.26	828
Patient 2	3.03	0.60	1792	3.01	0.63	188	2.60	0.29	12	2.58	0.24	43	3.56	0.96	1,545
Patient 3	2.80	0.48	573	2.92	0.49	169	2.53	0.20	30	2.60	0.30	47	3.31	0.80	1,038
Mean	2.87			3.02			2.62			2.62			3.55		
SD	0.13			0.10			0.11			0.06			0.24		
Control 1	3.27	0.90	1,473	3.56	1.10	568	3.15	0.74	255	2.98	0.63	255	4.19	1.65	1,128
Control 2	3.58	1.09	2,671	3.54	1.09	608	3.25	0.79	552	2.97	0.57	290	4.21	1.69	1,422
Control 3	2.78	0.45	610	2.92	0.54	277	2.75	0.38	133	2.64	0.29	171	2.88	0.57	247
Mean	3.21			3.34			3.05			2.87			3.76		
SD	0.40			0.36			0.27			0.20			0.76		

Research has investigated perpetrators of IPV, using MRI to identify changes in brain activity and structure that may influence the propensity for committing IPV, including evidence that reduced DMN activation suggests perpetrators do not view IPV-related decisions towards their partners as a moral conflict (Marín-Morales et al., 2020), a higher ratio of dorsal/ventral medial prefrontal cortex reactivity gradient is indicative of increased aggression in IPV perpetration (Chester and DeWall, 2019), perpetrators exhibit differential activation of the cingulate and prefrontal cortices in response to IPV imagery (Bueso-Izquierdo et al., 2016), and may have structural deficits in the amygdala (Zhang et al., 2013), with alterations in cortical gray matter thickness among perpetrators of IPV (Verdejo-Román et al., 2019). It is crucial to identify how we can improve the quality of care for existing and future survivors of IPV by increasing awareness and further emphasizing the importance of screening for TBI among at-risk individuals to improve health outcomes. In conclusion, injuries to the head, neck, and face, are strongly associated with TBI among IPV survivors. Early and accurate characterization of TBI in survivors of IPV may help guide treatment and relieve certain neuropsychological consequences for the survivors. Treatments specifically targeting TBI neurorehabilitation may be beneficial to survivors of IPV.

## Data Availability Statement

The datasets presented in this article are not readily available because it is small dataset with potentially identifiable information, thus it is not being shared. Requests to access the datasets should be directed to GK, gkk6@case.edu.

## Ethics Statement

The studies involving human participants were reviewed and approved by Cleveland Clinic IRB. The patients/participants provided their written informed consent to participate in this study.

## Author Contributions

GK conceived the idea, designed the study, data collection, and writing the results through the publication process. KW worked on designing the study, data collection, and writing. SEJ worked on designing, reviewing the images, and image analysis. MJL worked on the image analysis. SMR contributed to the study design. All authors contributed to the article and approved the submitted version.

## Funding

This publication was made possible by the US National Health Institutes (NIH) grant R01-LM012518 from the National Library of Medicine. This publication was also made possible in part by the Clinical and Translational Science Collaborative of Cleveland, KL2TR000440 from the National Center for Advancing Translational Sciences (NCATS) component of the National Institutes of Health and NIH Roadmap for Medical Research. Its contents are solely the responsibility of the authors and do not necessarily represent the official views of the NIH.

## Conflict of Interest

The authors declare that the research was conducted in the absence of any commercial or financial relationships that could be construed as a potential conflict of interest.

## Publisher’s Note

All claims expressed in this article are solely those of the authors and do not necessarily represent those of their affiliated organizations, or those of the publisher, the editors and the reviewers. Any product that may be evaluated in this article, or claim that may be made by its manufacturer, is not guaranteed or endorsed by the publisher.
